# Optimal cutoff value of basal anti-mullerian hormone in iranian infertile women for prediction of ovarian hyper-stimulation syndrome and poor response to stimulation

**DOI:** 10.1186/s12978-015-0053-4

**Published:** 2015-09-10

**Authors:** Malek Mansour Aghssa, Azam Manshadi Tarafdari, Ensieh Shahrokh Tehraninejad, Mohammad Ezzati, Maryam Bagheri, Zahra Panahi, Saeed Mahdavi, Mehrshad Abbasi

**Affiliations:** Vali-e-Asr Reproductive Health Research Center, Department of Obstetrics and Gynecology, Valiasr Hospital, Tehran University of Medical Sciences, 1419433141 Tehran, Iran; Department of Obstetrics and Gynecology, Washington Hospital Center, Washington, DC USA; Saeed Medical Laboratories, Tehran, Iran; Department of Nuclear Medicine, Valiasr Hospital, Tehran University of Medical Sciences, Tehran, Iran

**Keywords:** Assisted Reproductive Technology, Ovulation induction, Ovarian hyper-stimulation syndrome, Anti-mullerian hormone

## Abstract

**Aim:**

We intended to establish the threshold of Anti-Mullerian Hormone (AMH) for detection of Ovarian Hyper-Stimulation Syndrome (OHSS) and poor response to treatment in Iranian infertile women.

**Methods:**

Pre-stimulation menstrual cycle day-3 hormonal indices including basal AMH values were measured in 105 infertile women aged 32.5 ± 4.3 years. Patients underwent long GnRH agonist Controlled Ovarian Hyperstimulation (COH) in a referral infertility center (Tehran, Iran). The gonadotropin dose was determined based on the age and basal serum Follicular Stimulating Hormone (FSH) level. The IVF/ICSI cycles were followed and the clinical and sonographic data were recorded.

**Results:**

Sixteen cases developed OHSS. The prevalence of PCOS was higher in subjects with OHSS [62.5 % (38.8-86.2) vs. 17 % (9.2-24.9)]. The patients with OHSS had higher ovarian follicular count [23.7 (3.2) vs. 9.1 (0.5); p < 0.05], collected oocytes [13.5 (1.9) vs. 6.9 (0.5); p < 0.05] and AMH level [7.9 (0.7) vs. 3.6 (0.3); p < 0.05]. Basal AMH level and oocyte yields (but not age, BMI, and PCOS) correlated with occurrence of OHSS; and only the AMH levels were associated with poor ovarian response (oocytes yield ≤ 4). The optimal cutoff value for the prediction of OHSS was 6.95 ng/ml (area under the receiver operating characteristics curve: 0.86; CI: 0.78-0.95; sensitivity: 75 %; specificity: 84 %; odds ratio for occurrence of OHSS: 9 and p < 0.001). The optimal cut point to discriminate poor response (oocytes ≤4) was 1.65 ng/ml ( AUC : 0.8; CI: 0.69-0.91; sensitivity: 89 % specificity : 71 %; and OR = 23.8 and P value <0.001).

**Conclusions:**

Iranian women with basal AMH level > 6.95 ng/ml are at high risk of developing OHSS and those with AMH level < 1.65 ng/ml are poor responders.

## Background

Anti-Mullerian Hormone (AMH) is a granulosa cell derived hormone secreted from pre-antral and small antral follicles. AMH substantially inhibits the initiation of primordial follicle growth and contributes to normal folliculogenesis by enhancing the role of FSH in cyclic recruitment of follicles [1]. Clinically, AMH can serve as a reliable ovarian reserve marker [2] independent of gonadotropins levels [3]. A particularly helpful aspect of AMH, when used as an ovarian reserve marker, is that its serum levels remain relatively constant during normal menstrual cycles [4–6]. The reported variability during the menstrual cycles is not possibly clinically influential [7]. In 2002, Seifer et al*.* underscored the association of AMH levels with ovarian response to Controlled Ovarian Hyperstimulation (COH) [8]. The recent meta-analysis by Broer et al. highlighted 9 studies employing AMH to predict excessive responses during COH [9]. While the ability to predict excessive ovarian stimulation using basal AMH values is established, the optimal threshold of AMH to predict Ovarian Hyper-Stimulation Syndrome (OHSS) is controversial and subjected to this research.

## Methods

A total of 105 infertile couples undergoing COH enrolled in this study. They were visited in a private referral infertility center (Tehran, Iran) between March 2010 and February 2011. Subjects with any known systemic diseases or endocrine disorders including diabetes, hypothyroidism, hyper-prolactinemia, and those who were receiving levothyroxine or cabergoline were excluded. Data of the first attempt was recorded for the patients who underwent more than one IVF/ICSI cycle. The corresponding demographic and infertility related data of the participants were collected and the baseline pre-stimulation AMH, FSH, LH, testosterone, dehydroepiandrosterone sulfate, TSH, and prolactin plasma levels were measured on the third day of the previous menstrual cycle. Pre-stimulation cycle(s) was/were induced in those without regular menses with intramuscular injection of 100 mg progestron in oil (Iran Hormone, Tehran, Iran) and maintained with daily OCP from day 3 in those without history of regular menses after withdrawal bleed. Serum AMH was measured using ultrasensitive ELISA (Beckman-Coulter Ireland, Inc., Galway, Ireland) with functional sensitivity of 0.2 ng/ml and intra and inter-assay coefficient of variability of 8 and 12 %, respectively. Samples were centrifuged and the assays were done in serums after performing calibrations according to the manufacturer instructions. Samling of the hemolyzed samples were repeated and no particular strategy for handling Heterophile antibody was employed. The chief gynecologist of the center (*i.e.* MMA) selected the stimulation protocol, type, and dose of the gonadotropin and diagnosed and managed OHSS irrespective of patients’ participation in the study. The follicle count (≥14 mm) based on sonographic examination on the day of HCG administration, the number of retrieved oocytes, and the final outcome of the IVF/ICSI cycle were recorded.

### Controlled ovarian hyperstimulation protocol

Participants were recruited among those who underwent long GnRH agonist protocol irrespective of age, gonadotropin dosage, and history of OHSS or antral follicular count. From day 21 of the pre-stimulation menstrual cycle, the patient received a daily subcutaneous injection of Boucerelin acetate (Superfact, Hoecht AG, Frankfurt, Germany). The gonadotropin (GonalF, Serono, Switzerland) was added on day 2 or 3 of the IVF/ICSI cycle. Dosing was determined by the chief treating gynecologist of the center mainly based on the age and basal FSH levels (ranged between 150 and 225 IU/day). Dosage of gonadotropin was adjusted based on the degree of ovarian response in interval sonographic examinations (data not collected for this report). Patients were examined by daily trans-vaginal sonography starting on day 7 of stimulation. HCG (250 mgr; Ovitrelle, Merck, Serono) was injected subcutaneously when the sonographic examination showed a minimum of two 18 mm follicles. Oocyte pickup was performed from posterior vaginal fornix 34 to 36 hours after HCG administration. Embryo was transferred within 2 days of oocyte pickup and the patient received 400 mg of cyclogest (Alpharma, Barnstaple, UK) every 12 hours during the first 12 weeks of gestation and 2 mg of estradiol valereate every 12 hours for 2 weeks. Serum βHCG was measured 15 days after embryo transfer. Biochemical pregnancy was defined as βHCG value > 50 mIU/ml. Sonographic examination was performed 2 weeks later to confirm the presence of gestational Sac and then 2 weeks afterward to confirm viability of the embryo.

The OHSS related symptoms and signs, including abdominal distension and discomfort, nausea, vomiting, diarrhea, ascites, plural effusion, edema, oliguria, hypercoagulative state, serum creatinine of 1.0-1.5 mg/ml, hemoconcentration, and electrolyte imbalance were monitored. Suspected OHSS cases were examined by sonography to determine ovarian enlargement and ascites [10]. OHSS and the severity of the condition were defined according to the Navot et al. [11]. Those patients experiencing OHSS were managed by cycle cancellation, coasting, or freezing the embryo for future IVF cycles with or without additional cabergoline (dostinex) therapy.

The ethical committee of the Faculty of Medicine (Tehran University of Medical Sciences) approved the study and waved the need for written informed consent. The data was handled and analyzed anonymously.

### Statistics

Independent sample T-tests and Chi square tests were used to compare correspondingly the difference of continuous values and the prevalence of categorical variables between subjects with and without OHSS. A binary logistic regression model (enter method) was designed to study the multivariate correlation of OHSS with age, BMI, basal biochemical indices (including AMH), sonographic findings (follicle count), and clinical outcomes (retrieved oocytes). Additionally, a univariate general linear model (enter method) was employed to adjust the effect of covariates on the association of OHSS and AMH level. Finally, a receiver operating characteristics (ROC) curve was created to classify subjects with and without OHSS according to AMH levels. Two different approaches were assessed to determine the optimal cutoff value of AMH to classify OHSS: Youden index which is the maximum sensitivity - (1-specificity) and the shortest distance on the ROC from the optimal sensitivity and specificity [(1 - sensitivity)^2^ + (1 - specificity)^2^] [12]. Sensitivity, specificity, positive likelihood ratio [PLR; sensitivity / (1 - specificity)], and negative likelihood ratio [NLR; (1 - sensitivity) / specificity] of the cutoffs were also calculated [13]. A binary logistic regression model was also designed to predict poor response to stimulation with different variables including AMH level. The response to stimulation was defined poor ≤4 collected oocytes or good > 4 collected oocytes in this model.

The AMH values were not normally distributed (Kolmogorov-Smirnov’s P value <0.001) and were positively skewed. For the parametric tests the square root of the AMH values was generated and employed (Kolmogorov-Smirnov’s P value of transferred data = 0.39).

## Results

The subjects’ characteristics are shown in Table 1. Basal AMH level as well as ovarian follicle and collected oocytes counts but not age and BMI were higher in the subjects with OHSS. The mean basal AMH value of the 105 studied cycles was 4.2 ng/ml [SD: 3.3, median: 3.5, inter-quartile range: 1.4-7.0, range: 0.05-15.0]. Sixteen patients presented with moderate or severe OHSS (15.2 %) out of whom 4 cycles were canceled, oocytes were collected for subsequent IVF attempts in 7 cases (with additional dostinex therapy in one patient), ovulation induction was coasted in 2 patients (with additional dostinex therapy in one patient), and two subjects were treated with dostinex alone. Two cases with severe OHSS were managed in the hospital setting. Forty-two (40 %; CI: 30.6-49.4 %) IVF/ICSI cycles led to clinical pregnancies and 37 (35.2 %; CI: 26.1-44.4 %) live births. The subjects with OHSS had a clinical pregnancy rate of 3/16 in the studied cycles with only 2 live births both in subjects treated with dostinex alone. In 4 patients (25 %) the OHSS occurred early and severely enough to prevent oocyte collection and 2 patients, out of 89 subjects (2.2 %), without OHSS had no oocytes yield.

**Table 1:** The characteristics of the subjects with and without ovarian hyper-stimulation syndrome

	Subjects without OHSS *n* = 89	Subjects with OHSS *n* = 16	Total *n* = 105
Age (yrs)	32.8(0.5)	31(0.9)	32.6(0.4)
BMI (kg/m^2^)	24.8(0.5)	25.8(1)	25(0.4)
Duration of infertility (yrs)	6.1(0.5)	6.7(0.9)	6.2(0.4)
Duration of stimulation (days)	10.6(0.2)	9.7(0.5)	10.5(0.2)
Number of follicles (HCG day)	9.1(0.5)	23.7(3.2)	11.3(0.8)†
Number of retrieved oocytes	6.9(0.5)	13.5(1.9)	7.7(0.5)†
Basal Anti-mullerian Hormone	3.6(0.3)	7.9(0.7)	4.3(0.3)†
Basal Luteinizing Hormone	7.6(0.5)	7.8(1.2)	7.6(0.4)
Basal Follicle stimulating Hormone	7.8(0.5)	5.1(0.5)	7.4(0.4)
Thyroid stimulating hormone	2.5(0.2)	1.9(0.3)	2.4(0.2)
Basal Prolactin	88.9(23.6)	58(18.8)	84(20.1)
Basal Testosterone	120.6(16)	99.6(30)	117.3(14.3)
Basal DHEAS	163.9(19.7)	124.8(22.4)	157.5(16.9)
Polycystic ovary syndrome	17(9.2-24.9)	62.5(38.8-86.2)	23.8(15.7-32)†
Clinical pregnancy	44.3(33.9-54.7)	20(−0.2-40.2)	40(30.6-49.4)

Data are mean or percentages and standard error from the mean or 95 % confidence intervals in the parentheses.

† indicates significant difference between the values of subjects with and without ovarian hyper-stimulation syndrome (T-test or Chi squared test; p < 0.05).

Twenty-five subjects (23.8 %; CI: 15.7-32.0 %) had PCOS with higher prevalence of OHSS (40 % vs. 7.6 %; p < 0.001), younger age [30.5(0.7) vs. 33.3 (0.5); p = 0.005] and higher basal AMH levels [7.1(0.7) vs. 3.3(0.3); p < 0.001]. Their pregnancy rate, however, was not statistically different from other patients [28(10.4-45.6) vs. 44.3(33.3-55.3); p = 0.1].

The binary logistic regression model was designed with age, BMI, number of previous OSCs, PCOS, FSH level, number of retrieved oocytes, and basal AMH level (square root) as independent variables to predict the OHSS (dependent variable). The number of retrieved oocytes [OR: 1.3(95 CI: 1.1-1.6), Wald: 8.0, and P = 0.004] and basal AMH value [OR: 5.4 (95 CI: 1.1-27.9), Wald: 4.1, and P = 0.04] (but not PCOS, age, and BMI) were significant independent predicting factors of OHSS. The square root of basal AMH levels were moderately correlated with the number of retrieved oocytes (r = 0.5; p < 0.001). Another model was defined with follicle count instead of collected oocytes count (considering significant co-linearity). In this model, in addition to AMH level the follicle count was significantly associated with OHSS [OR: 1.5 (95 CI: 1.1-1. 9), Wald: 8.9, and P = 0.003]. A binary logistic regression model was defined with independent variables identical to those of the previous models excluding collected oocyte or follicle counts. AMH level (square root) was the only predictor of the response to stimulation [OR: 0.25 (95 CI: 0.11-0.59), Wald: 10.3, and P = 0.001].

The total administered gonadotropin dose (but not total days of stimulation) correlated inversely with the OHSS occurrence (OR for every additional 75 international unit = 0.8, CI: 0.7-0.9; p = 0.002).

**Fig. 1:**
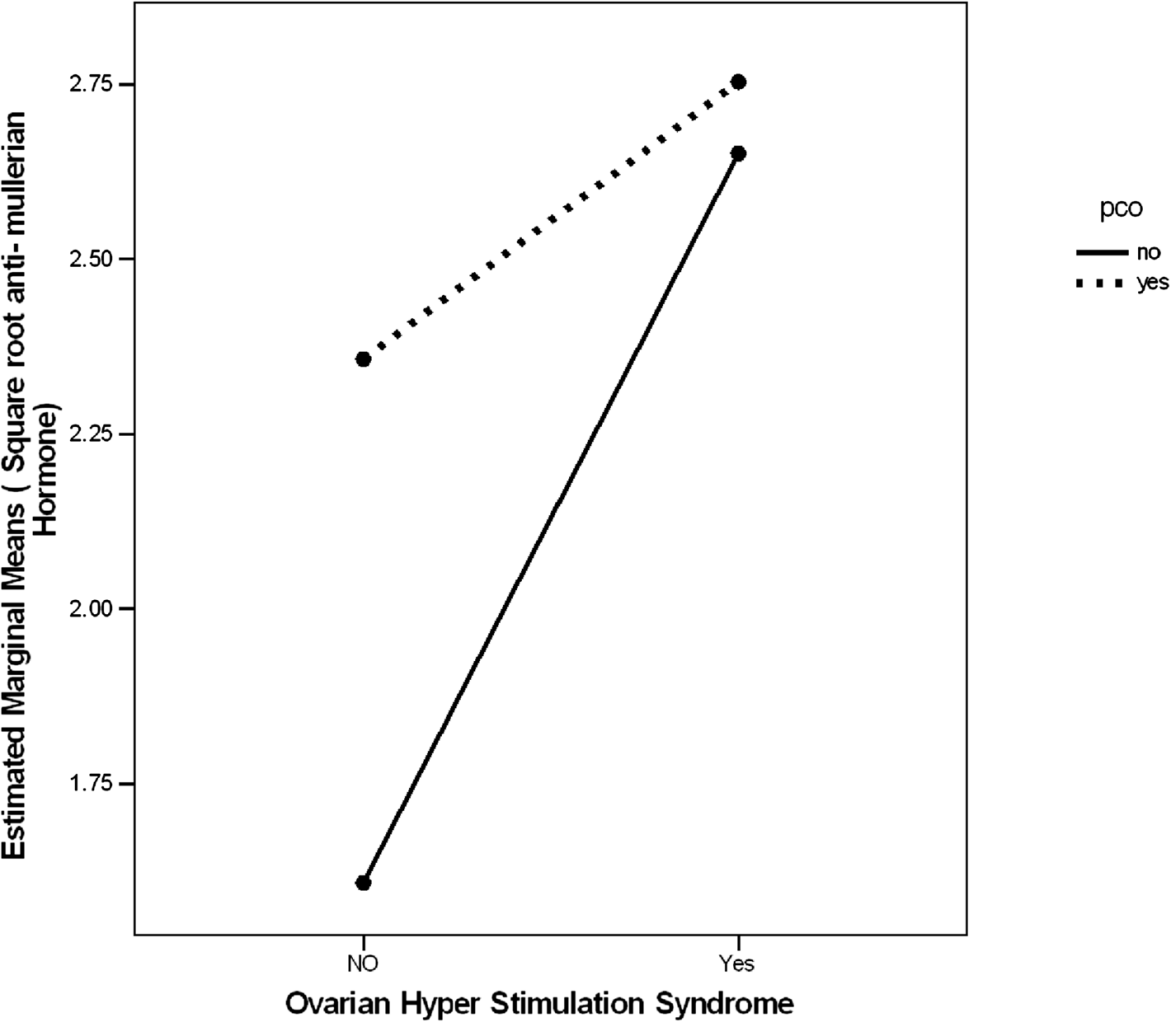
Age adjusted anti-mullerian hormone is higher in those with consequent ovarian hyper-stimulation syndrome in patients with and without polycystic ovary syndrome

After adjustment for the effect of age, BMI, and PCOS; the AMH values were higher in the subjects with consequent OHSS than in those without OHSS [7.7 (0.7) ng/ml vs. 4.7(0.4) ng/ml; df = 5, F (11.9) = 14.2, eta^2^ = 0.1; P = 0.001]. No significant interaction effect was detected for the presence of PCOS on the association of AMH levels and OHSS [F (11.9) =2.4 and P = 0.1; Fig. 1].

**Fig. 2:**
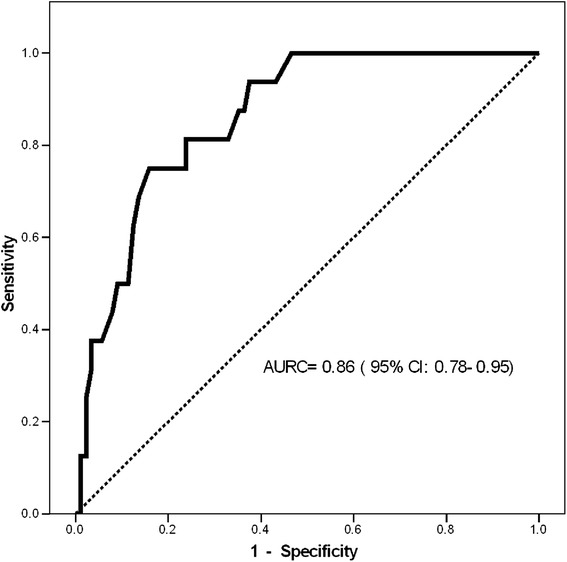
The Receiver Operating Characteristics (ROC) curves of basal anti-mullerian hormone values to predict ovarian hyper-stimulation syndrome. AURC: Area Under the ROC Curve

**Fig. 3:**
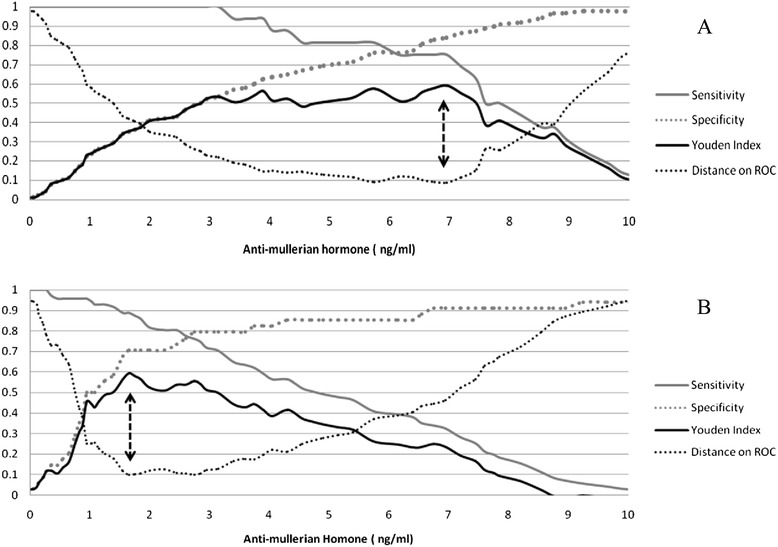
Sensitivity, specificity, Yuden index and distance to the optimal point on the ROC curve for the basal anti-mullerian hormone levels to predict ovarian hyper-stimulation syndrome (panel **a**) and poor response to the IVF cycle (panel **b**). Arrows indicate the most effective threshold value of AMH (6.95 and 1.65 ng/ml for ovarian hyper-stimulation syndrome and poor response to ovarian stimulation, respectively) corresponding to both maximum Youden index and shortest distance on the ROC curve

AMH levels classified subjects with and without OHSS with an area under the ROC curve (AUC) of 0.86 (0.78-0.95; Fig. 2); the best AMH cutoff value to predict OHSS was 6.95 ng/ml (sensitivity: 75 %, specificity: 84 %, PLR: 4.7, NLR: 0.3; Fig. 3, panel a). Patients with AMH values higher than 6.95 ng/ml experienced a higher frequency of OHSS (5.1 % vs. 46.2 %; OR = 9, CI: 1.3-59.7, Chi^2^ P value < 0.001). In subjects without OHSS, those with AMH values over 6.95 (n = 14) compared to those with AMH levels below 6.95 received significantly smaller gonadotropine doses per day [157 (17) IU vs. 197 (46); p < 0.005] with higher collected oocytes [9.4 (1.0) vs. 6.4 (0.5); p < 0.05] and the equal follicle count [11.1 (0.7) vs. 8.7 (0.7); p < 0.067]. The cutoff value of AMH with the best prediction of poor response to controlled ovarian stimulation (oocytes ≤4) was 1.65 ng/ml with AUC of 0.8 (0.69-0.91) and sensitivity and specificity of 89 % and 71 %, respectively (Fig. 3, panel b). A poor response rate in those with an AMH value below and above the cut point (1.65) was 75 % and 13.7 %, respectively (OR = 23.8, CI: 6.0-94.1, Chi^2^ P value <0.001). The cut points for detection of OHSS and poor response were substantially the same after exclusion of subject with PCOS (data not shown).

No significant association was found between basal AMH level and the outcome of the IVF/ICSI procedure (clinical pregnancy vs. failed cycle).

## Discussion

Our results indicate an association between extreme AMH levels and OHSS and poor response to controlled ovarian stimulation independent of the effect of age, BMI, and a history of PCOS. According to our findings, subjects with an AMH level <1.65 (almost the lower quartile of AMH values) are more likely to be poor responders to ovarian stimulation (post-test probability: 75 %; OR = 23) and those with AMH levels >6.95 (almost the upper quartile) are at a higher risk for OHSS (post-test probability: 46 %; OR = 9). With subclass analysis of the administered gonadotropin dose, we suggest that milder ovarian stimulation in high risk patients has no detrimental effect on the COH main outcome (retrieved oocyte count). Thus, we believe that based on the proposed cut point of basal AMH levels (i.e. 6.95; roughly the upper quartile), reduced stimulation would be a safe and the reasonable method of preventing OHSS and its consequences. This approach may decrease the severe OHSS cases with its unfavorable consequences including hospitalization and cycle cancellation [14]. After adjustment for AMH levels, other pre-stimulation variables including age, BMI, and PCOS did not correlate with OHSS or poor response to COH.

Our optimal cutoff value of AMH for the prediction of OHSS is higher than the cut points suggested by previous studies (i.e. 1.6 ng/ml [15], 2.1 ng/ml [16], 3.4 ng/ml [17], 3.5 ng/ml [18, 19] and 4.8 ng/ml [20]). Our results are in line with the result of La Marca et al. [4] with cutoff value of 7 ng/ml (75^th^ percentile) for the classification of exaggerated responses. The threshold value of the study by Lee et al. was numerically lower but again it was the 75^th^ percentile of the AMH values of the studied population. Ebner et al. [21] also reported the best ovarian response was achieved when the basal AMH value was between the 25^th^ and 75^th^ percentiles with reduced oocyte quality of patients in the top quartile (AMH level > 4.5 ng/ml). The cutoff point suggested by Nardo et al. (3.5 ng/ml) is presumably the upper quartile of non-PCOS normal responders to COH. The differences between the AMH thresholds for higher OHSS risk noted in this study as compared to other studies may be explained by the differences between the studied populations (age, threshold for treatment of infertility, or frequency of PCOS), the definition of OHSS, and the different AMH measurement methods. Roughly - also not limited by the remarkable difference between the AMH readings of two older ELISA kits i.e. DSL and Immunotech - the patients with upper quartile AMH values are at high risk for exaggerated ovarian response [22]. Generally there is good correlation between the new (i.e. Beckman-Coulter) and old AMH assay kits [23].

In our study 15 % of cases had moderate to severe OHSS. Moderate OHSS is reported in 1 to 14 % of subjects with less than 1 % severe OHSS cases [24]. We had higher frequency of diagnosed OHSS compared to the studies by Lee et al. (8 %), Nardo (10 %) and Aramvit et al. (12 %). Nevertheless, the prevalence of severe cases leading to hospitalization was relatively low [25]. The risk of excessive ovarian response is a function of the COH protocol and the health characteristics of the participants (including age and the prevalence of PCOS). One may expect to encounter less exaggerated responses in the short COH protocols and in populations with lower PCOS. The prevalence of PCOS was high among the infertile population of this study (i.e. 23.8 %). The prevalence of PCOS among subjects undergoing COH varies from 4 to 22 % [17, 19, 26]. The risk of OHSS in our study was dramatically high in patients with PCOS (40 %), which is consistent with the study by Aramvit et al. in which about 45 % of the PCOS subjects experienced OHSS [26]. Interestingly the incidence of OHSS associated with AMH high levels independent of the diagnosis of PCOS. Also after adjustment for the effect of AMH there was no correlation between PCOS and OHSS.

The clinical pregnancy rate was comparable and rather high in our study (40 %) as compared with the results of Nardo et al. (24 %) [19], Kini et al. (34 %) [27], and Lee et al. (41 %) [17]. Relatively higher pregnancy rates accompany higher exaggerated ovarian stimulation rates possibly due to stronger protocols or possibly higher prevalence of younger PCOS patients.

Our study suffers from certain flaws: above all the OHSS was mainly diagnosed based on the decision of the chief researcher who decided also for the future treatment of the patients. This reduces the extend the results could be extrapolated. The sample size of the study was also rather small, hence the conclusion regarding the pregnancy rate after assisted reproduction cycles should be interpreted with caution; however, this result adds information regarding the ethnic group being studied here. Also we employed long down-regulated protocol because at the time of study antagonist agents were unavailable to us. Lastly, since the measurement of AMH by Gen II assay is subject to the complement interference which may overestimate or underestimate the actual AMH values, further studies with the use of new protocols even in the already studied populations are justified [28].

## Conclusions

We suggest that infertile patients undergoing COH with top and low quartile basal AMH values are at high risk for OHSS and poor ovarian response, respectively. Reduced stimulation dosage in high risk subjects for OHSS had no detrimental effect on the final outcome. Our study indicated that AMH was a superior predictor to traditional factors including age, BMI, and PCOS. Also considering that AMH is available before the stimulation, it is superior to the number of oocytes yielded.
